# Evaluation of the Retinal Vasculature in Hypertension and Chronic Kidney Disease in an Elderly Population of Irish Nuns

**DOI:** 10.1371/journal.pone.0136434

**Published:** 2015-09-01

**Authors:** Amy McGowan, Giuliana Silvestri, Evelyn Moore, Vittorio Silvestri, Christopher C. Patterson, Alexander P. Maxwell, Gareth J. McKay

**Affiliations:** 1 Centre for Public Health, Queen’s University Belfast, Belfast, Northern Ireland; 2 Centre for Experimental Medicine, Queen’s University Belfast, Belfast, Northern Ireland; 3 Department of Ophthalmology, Royal Victoria Hospital, Belfast, Northern Ireland; Swinburne University of Technology, AUSTRALIA

## Abstract

**Background:**

Chronic kidney disease (CKD) and hypertension are global public health problems associated with considerable morbidity, premature mortality and attendant healthcare costs. Previous studies have highlighted that non-invasive examination of the retinal microcirculation can detect microvascular pathology that is associated with systemic disorders of the circulatory system such as hypertension. We examined the associations between retinal vessel caliber (RVC) and fractal dimension (D_*F*_), with both hypertension and CKD in elderly Irish nuns.

**Methods:**

Data from 1233 participants in the cross-sectional observational Irish Nun Eye Study (INES) were assessed from digital photographs with a standardized protocol using computer-assisted software. Multivariate regression analyses were used to assess associations with hypertension and CKD, with adjustment for age, body mass index (BMI), refraction, fellow eye RVC, smoking, alcohol consumption, ischemic heart disease (IHD), cerebrovascular accident (CVA), diabetes and medication use.

**Results:**

In total, 1122 (91%) participants (mean age: 76.3 [range: 56–100] years) had gradable retinal images of sufficient quality for blood vessel assessment. Hypertension was significantly associated with a narrower central retinal arteriolar equivalent (CRAE) in a fully adjusted analysis (*P* = 0.002; effect size = -2.16 μm; 95% confidence intervals [CI]: -3.51, -0.81 μm). No significant associations between other retinal vascular parameters and hypertension or between any retinal vascular parameters and CKD were found.

**Conclusions:**

Individuals with hypertension have significantly narrower retinal arterioles which may afford an earlier opportunity for tailored prevention and treatment options to optimize the structure and function of the microvasculature, providing additional clinical utility. No significant associations between retinal vascular parameters and CKD were detected.

## Introduction

Chronic kidney disease (CKD) is a growing global public health concern, with associated morbidity, premature mortality and substantial healthcare costs [1]. CKD rates are predicted to rise considerably over the next 20 years [2]. The number of individuals within the US population aged 30 years and over is predicted to exceed 204 million by 2020 and almost 225 million by 2030, with an estimated 28 million and 38 million adults affected by CKD in 2020 and 2030 respectively [2]. Associated health care costs and quality-of-life concerns will increase accordingly, stimulating further efforts to detect and slow the onset and progression of CKD.

CKD is associated with an increased risk of cardiovascular events, hospitalization and death [3]. CKD may accelerate atherosclerosis progression by a variety of mechanisms including increased oxidative stress [4]. Hypertension is both a well-established cause and a consequence of CKD [5, 6], and is the second leading cause of end-stage renal disease in the United States [7]. Controlling hypertension helps to delay CKD progression, indicating that blood pressure is a modifiable environmental risk factor for CKD [8, 9]. CKD prevalence is low in younger adults but increases significantly with advancing age [10].

The retinal vasculature is accessible to direct and repeated non-invasive assessment offering a unique opportunity to study subtle, early microvascular variation prior to clinically significant disease. Recent advances in both digital retinal photography and imaging technology have enabled better characterization of multiple retinal parameters. Assessment of global geometrical vascular features such as fractal dimension and tortuosity can provide novel measurements that may improve our understanding of microvascular disease processes [11–15]. Specifically, detection of variation in retinal microvascular parameters may allow improved disease risk stratification at earlier time points in disease progression [16, 17].

Previous studies have demonstrated an association between retinal arteriolar narrowing and hypertension [18–29], with some studies also reporting association between widening of the retinal venules and elevated blood pressure [22, 30–32]. Further support for these associations was provided in a recent meta-analysis of 10,229 subjects that concluded that both narrower retinal arterioles and wider venules were independently associated with an increased risk of hypertension, highlighting the importance of microvascular remodelling in the pathogenesis of hypertension [28].

Both the retinal and renal microvasculature systems share comparable anatomical and physiological properties [33–34]. The associations between novel retinal vascular changes and CKD have not been clearly established [35–45]. Cross-sectional studies have shown associations between narrower retinal arterioles and reduced fractal dimension with lower eGFR but interestingly not with CKD [39]. In contrast, others have reported significant venular dilatation in association with CKD in persons with and without diabetes [41]. Furthermore, others have reported significant retinal arteriolar narrowing in association with CKD [42]. In prospective studies, the MESA study reported an association between arteriolar narrowing and CKD in white ethnicities only [38], although others failed to find an association between vessel caliber and CKD [36, 44].

As such, a clearer understanding of the underlying pathogenic mechanisms and risk predictors associated with CKD could enable further development of appropriate preventive and therapeutic measures.

In this study, we sought to examine the relationship between specific retinal vascular parameters, hypertension and CKD status using cross-sectional data from the Irish Nun Eye Study (INES) which included 1233 well-characterized white female participants aged 56–100 years.

## Materials and Methods

### Study Population

In brief, the Irish Nun Eye Study (INES) was a cross-sectional observational study of eye health in white Irish nuns selected from convents across Ireland, with recruitment between 2007 and 2009 [46]. The study was designed to investigate the prevalence of age-related macular degeneration (AMD) in a population with a restricted lifestyle to examine the relationship between light exposure and AMD. A restricted lifestyle or monastic rule is a set of restrictions that governs behavior in terms of material possessions, emotional and physical attachment, maintenance of a daily structured religious life of abstinence and prayer with dietary and lifestyle limitations. Contact was made with 152 convents, of which 126 (82.9%) agreed to participate. One thousand, five hundred nuns in these convents were invited to participate in the study and 1233 agreed (82.2%). Those who did not participate tended to be ill or unavailable on the day of examination. The inclusion criteria mandated participants to be of Irish descent, aged over 55 years and have lived in a convent for at least 25 years. There were no specific exclusion criteria. In order to maximize recruitment and minimize disruption to participant routines, all examinations were carried out within the community. The study was approved by the Institutional Review Board and the Office for Research Ethics Committee Northern Ireland. Informed written consent was obtained from all participants prior to participation. Demographic data were obtained from interviews by a trained field worker using a structured questionnaire. Relevant information collected included history of cigarette smoking and alcohol use, medication usage and disease status (presence or absence). This study was specific to one ethnicity as only white Irish nuns were included.

### Anthropometric and Blood Pressure Measurements

Blood pressure was measured once in a seated position with an oscillometric blood pressure aneroid sphygmomanometer (Speider and Keller) after the questionnaires had been completed. Mean arterial blood pressure (MABP) was calculated as one third of the systolic (SBP) plus two thirds of the diastolic blood pressure (DBP). Hypertension was determined if systolic blood pressure ≥ 140 mm Hg or diastolic blood pressure ≥ 90 mm Hg or if on antihypertensive medication. Classification of diabetes was determined by self-report. Height, weight, and waist circumference were measured and body mass index (BMI) calculated as weight (in kilograms) divided by height (in meters) squared.

### CKD Characterization

Estimated glomerular filtration rate (eGFR) was calculated from serum creatinine measurements using the CKD-Epi (Chronic Kidney Disease Epidemiology Collaboration) equation [47]. Those individuals with an eGFR<60 mL/min/1.73 m^2^ were classified as having CKD, and those with an eGFR≥60 mL/min/1.73 m^2^ were classified as not having CKD.

### Ocular Examination and Retinal Photography

Each individual underwent a comprehensive ophthalmic examination. Medical and ophthalmic questionnaires covered areas such as medical and ocular history. Refractive error was recorded either from a recent prescription or from the participant’s glasses. Where glasses were not available, corrected visual acuity was achieved by pinhole correction; refraction was not carried out. Retinal findings were recorded by stereoscopic retinal imaging using the Nidek AFC 210 digital camera. Fields 1 and 2 were captured following dilation of the pupils with 1% tropicamide.

### Retinal vessel caliber assessment

**Fig 1:**
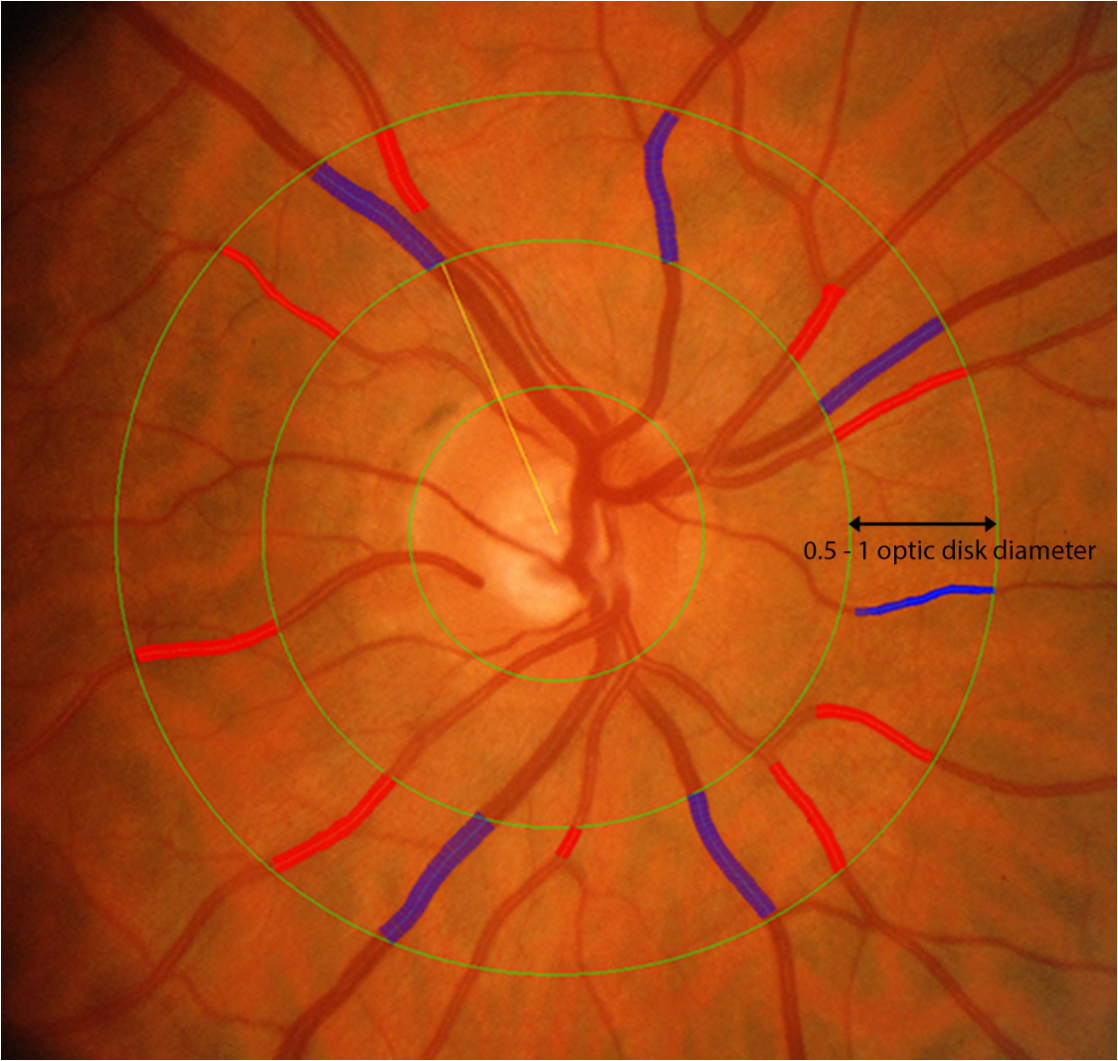
Retinal vascular calibre measurement. Retinal arteriolar and venular calibers were summarized as central retinal arteriolar (CRAE) and the central retinal venular (CRVE) equivalent respectively from digital retinal fundus images using the Interactive Vessel Analysis software (IVAN, University of Wisconsin, US). Arterioles are in red and venules are in blue.

Retinal arteriolar and venular calibers were measured using Interactive Vessel ANalysis software (IVAN; University of Wisconsin, Madison, WI) according to a standardized protocol for all retinal vessels located between a half and one disc diameter distance from the optic disc margin in the digitized image (Fig 1). The revised Knudston-Hubbard formula [48] was used to summarize these measurements as CRAE (Central Retinal Arteriolar Equivalent) and CRVE (Central Retinal Venular Equivalent), which represent the average caliber of the arterioles and venules in each eye examined. A single trained grader (AMG), blinded to participants characteristics, conducted all retinal measurements. Reproducibility of retinal vascular measurements was high with intra-grader reliability assessed in 200 randomly selected retinal photographs and an intra-class correlation coefficient (95% confidence interval) calculated as 0.975 (0.967–0.981) for CRAE and 0.993 (0.990–0.994) for CRVE, respectively. A high correlation between the right and left eyes in retinal vascular measurements has been reported elsewhere [49]. Data from the right eye was used and when unavailable, was replaced by the left eye.

### Retinal vascular fractal dimension assessment

**Fig 2:**
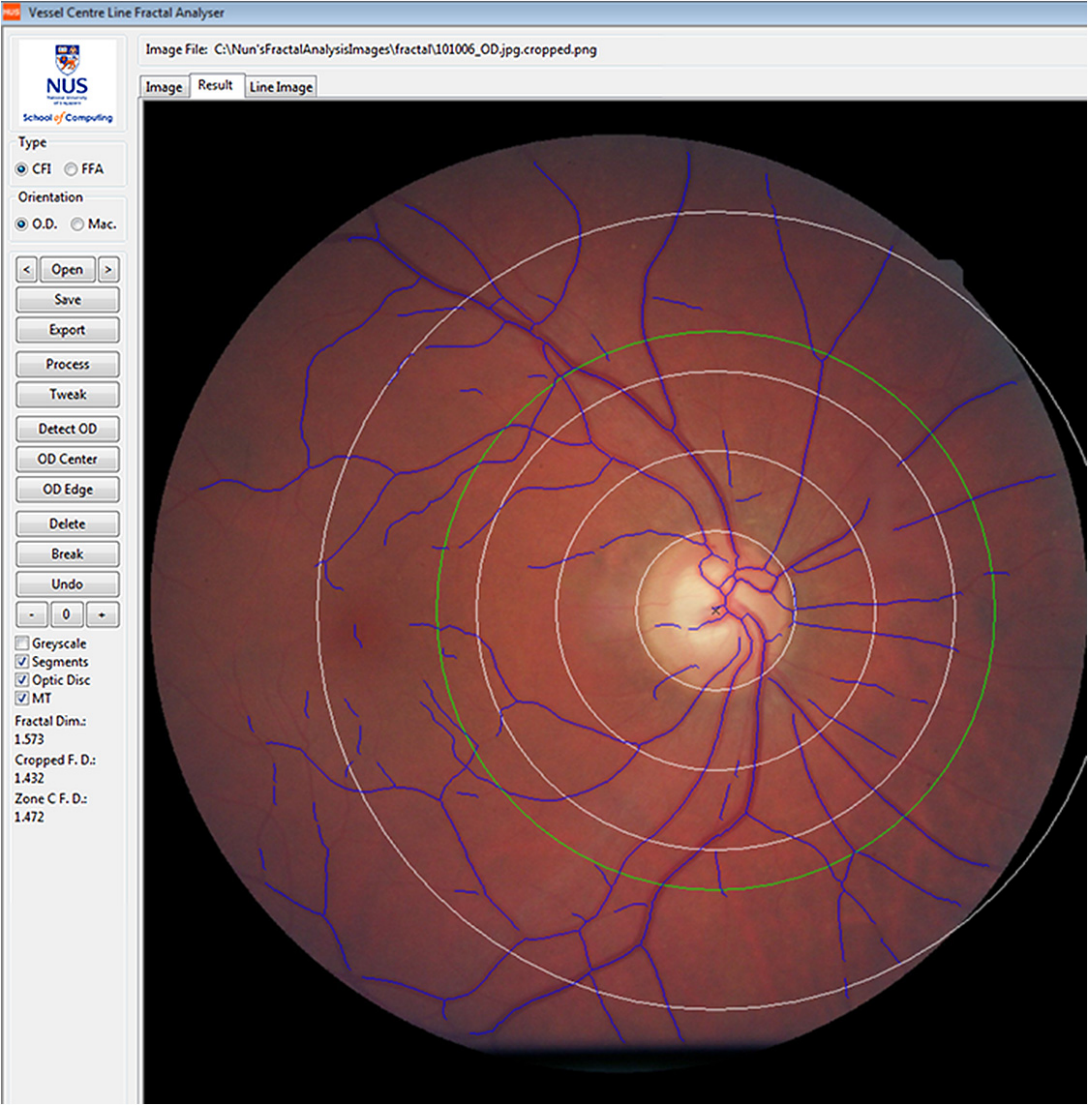
Retinal vascular fractal dimension measurement. The upper image illustrates a retinal fundus image and skeletonized line tracing of an eye with a low fractal dimension and less complex (more rarefied) branching pattern; the lower retinal fundus image and skeletonized line tracing illustrates a higher fractal dimension and a more complex (dense) branching pattern.

Fractal analysis was performed using digital retinal images centered on the optic disc. A single blinded trained grader (AMG) used a computer-based program [SIVA-FA, software version 1.0, School of Computing, National University Singapore] for measurement of fractal dimensions (D_*F*_) according to a standardized protocol [50]. Briefly, the optic disc was automatically detected by the software which identified the edges of the optic nerve head. The fractal dimension of the retinal vasculature was calculated within a predefined circular area from 0.5 to 2 disc diameters (D_disc_) away from the optic disc margin (Fig 2). The software performs automated skeletonized vessel tracing that does not differentiate between the arterioles and venules. Artefacts generated from choroidal vessels, peripapillary atrophy, pigmentary abnormalities and reflection from the nerve fibre layer were identified and manually erased. The software computed a D_*F*_ from the refined skeletonized vessel tracing using the box-counting method which involves drawing predetermined size boxes which overlay the structures of interest calculating a fractal value [51]. These values represent a “global” summary measure of the whole branching pattern of the retinal vascular tree with larger values indicative of a more complex branching pattern [52].

### Statistical Analysis

The independent samples t test and chi-squared test were used to compare the characteristics of those in the study with and without hypertension, and with and without CKD. Quantitative retinal vascular caliber (CRAE and CRVE) and fractal dimension were assessed as continuous variables.

Multiple linear regression models were used to analyze the association of retinal vascular parameters with both hypertension and CKD in unadjusted analyses (Model 1) and adjusted analyses (Models 2, 3 and 4). The minimally adjusted model included covariates for refractive error, age, BMI, smoking, alcohol consumption (yes/ no), diabetes mellitus status, ischemic heart disease (IHD), cerebrovascular accident (CVA) and CKD status (Model 2). The model was not adjusted for gender as all participants were female. Model 3 included the covariates from the minimally adjusted Model 2 and, in addition, medications used at a frequency >5% within the cohort (ACE [angiotensin converting enzyme] inhibitors, aspirin, beta blockers, calcium channel blockers, corticosteroids, diuretics, nonsteroidal anti-inflammatory drugs [NSAIDs] and statins). Model 4 included covariates from Model 3 and, in addition, the fellow vessel (venule or arteriole) caliber (i.e. CRAE as a covariate in the analysis of CRVE and vice versa) as suggested previously [53].

A *P* value < 0.05 was regarded as statistically significant. All statistical analyses were performed using IBM SPSS Statistics version 21 (IBM Corp., Armonk, NY).

## Results

In total, gradable retinal images of sufficient quality for vessel assessment were available in 1122 (91%) of the 1233 participants. Images were not available for 111 participants, mainly as a consequence of difficulties with image acquisition due to postural complications with the elderly participant, poor pupillary dilation, the presence of an artificial eye or an out of focus image. As previously reported (41), participants with missing retinal vascular parameter data (n = 111) were significantly older and more likely to have moderate to severe cataract (resulting in poor quality images) than those with retinal vascular parameter data available (P<0.001); 11 had no retinal images captured and 60 had macula-centered images only, which were not amenable to direct measurement comparisons using the IVAN software. The mean age of the 1122 participants included was 76.3 years (range: 56–100 years).

CRAE and CRVE were normally distributed, with means and standard deviations (SD) of 120.4 (12.6) μm and 169.0 (18.3) μm, respectively. Hypertension status was categorized as no hypertension (n = 454) and those with self-reported and/or clinically diagnosed hypertension (n = 667). The summary statistics for those with and without hypertension are displayed in Table 1. Those participants with hypertension were significantly older, with higher BMIs, greater MABP, were more likely to have IHD and CKD, and use ACE inhibitors, aspirin, beta-blockers, calcium channel blockers, diuretics, and statins (*P <* 0.001).

**Table 1:** Summary statistics of participants included for retinal vessel assessment by hypertension status.

Characteristic	With hypertension, n = 667	No hypertension, n = 455	*P*
Age, years Mean (SD)	77.8 (7.7)	74.0 (8.0)	**<0.001**
Body Mass Index, kg/m^2^ Mean (SD)	25.1 (5.2)	23.9 (4.8)	**<0.001**
MABP, mmHg Mean (SD)	96.8 (9.5)	86.0 (8.0)	**<0.001**
Ever smoked, n (%)	29 (4)	20 (4)	0.97
Any alcohol consumption^✝^, n (%)	44 (8)	33 (9)	0.57
Ischemic Heart Disease, n (%)	101 (15)	19 (4)	**<0.001**
Cerebrovascular accident, n (%)	25 (4)	11 (2)	0.22
Diabetes mellitus, n (%)	24 (4)	10 (2)	0.18
Chronic kidney disease, n (%)	410 (65)	213 (50)	**<0.001**
ACE inhibitors*, n (%)	121 (18)	13 (3)	**<0.001**
Aspirin*, n (%)	281 (42)	88 (19)	**<0.001**
Beta blockers*	167 (25)	25 (5)	**<0.001**
Calcium channel blockers*, n (%)	126 (19)	16 (4)	**<0.001**
Corticosteroids*, n (%)	34 (5)	28 (6)	0.45
Diuretics*, n (%)	208 (31)	32 (7)	**<0.001**
NSAIDs*, n (%)	29 (4)	24 (5)	0.47
Statins*, n (%)	303 (45)	142 (31)	**<0.001**
Central retinal arteriolar equivalent, μm (SD)	118.9 (12.4)	122.5 (12.5)	**<0.001**
Central retinal venular equivalent, μm (SD)	168.1 (18.2)	170.3 (18.5)	**0.04**
Fractal Dimension, D_*F*_	1.413 (0.062)	1.427 (0.062)	**<0.001**

SD: standard deviation; MABP: mean arterial blood pressure (one third of the systolic blood pressure plus two thirds of the diastolic blood pressure); ACE: angiotensin converting enzyme; NSAIDs: nonsteroidal anti-inflammatory drugs.

*Medications with a frequency >5%.

^✝^Data on alcohol consumption was only available in 941 participants who completed a food frequency questionnaire.

CKD status was dichotomized on the basis of eGFR, i.e. participants with CKD had an eGFR<60 mL/min/1.73 m^2^ (n = 623), and those without CKD were characterized as participants with an eGFR≥60 mL/min/1.73 m^2^ (n = 437). The summary statistics according to CKD status are presented (Table 2). Participants with CKD were significantly older (*P <* 0.001), with higher BMI (*P* = 0.03), greater MABP (*P* = 0.04), were more likely to be hypertensive (*P <* 0.001), with IHD (*P* = 0.005) and diabetes mellitus (*P* = 0.03), and appropriately medicated (ACE inhibitors, *P* = 0.001; aspirin, *P <* 0.001; beta-blockers, *P* = 0.005; diuretics, *P <* 0.001; statins, *P* < 0.001).

**Table 2:** Summary statistics of participants included for retinal vessel assessment by CKD status.

Characteristic	With CKD, n = 623	No CKD, n = 437	*P*
Mean Age, years (SD)	78.3 (7.6)	73.8 (7.9)	**<0.001**
Mean Body Mass Index, kg/m^2^ (SD)	24.9 (5.2)	24.2 (4.8)	**0.03**
Mean MABP, mmHg (SD)	92.9 (9.8)	91.5 (11)	**0.04**
Ever smoked, n (%)	24 (4)	22 (5)	0.35
Any alcohol consumption^✝^, n (%)	41 (7)	33 (9)	0.34
Ischemic Heart Disease, n (%)	81 (13)	33 (8)	**0.005**
Cerebrovascular accident, n (%)	24 (4)	11 (3)	0.23
Diabetes mellitus, n (%)	26 (4)	8 (2)	0.03
Hypertension, n (%)	410 (66)	221 (51)	**<0.001**
ACE inhibitors*, n (%)	90 (14)	35 (8)	**0.001**
Aspirin*, n (%)	247 (40)	103 (24)	**<0.001**
Beta blockers*	124 (20)	58 (13)	**0.005**
Calcium channel blockers*, n (%)	84 (13)	51 (12)	0.38
Corticosteroids*, n (%)	28 (4)	29 (7)	0.13
Diuretics*, n (%)	156 (25)	64 (15)	**<0.001**
NSAIDs*, n (%)	23 (4)	26 (6)	0.09
Statins*, n (%)	274 (44)	146 (33)	**<0.001**
Central retinal arteriolar equivalent, μm (SD)	120.0 (12.6)	120.8 (12.4)	0.27
Central retinal venular equivalent, μm (SD)	168.9 (18.7)	169.2 (17.9)	0.81
Fractal Dimension, D_*F*_	1.417 (0.061)	1.423 (0.063)	0.16

SD: standard deviation; MABP: mean arterial blood pressure (one third of the systolic blood pressure plus two thirds of the diastolic blood pressure); ACE: angiotensin converting enzyme; NSAIDs: nonsteroidal anti-inflammatory drugs.

*Medications with a frequency >5%.

^✝^Data on alcohol consumption was only available in 941 participants who completed a food frequency questionnaire.

### Hypertension

In an unadjusted analysis, individuals with hypertension had significantly narrower CRAE (*P <* 0.001; effect size = -3.62 μm; CI: -5.11, -2.14) compared to those without hypertension (Table 3). Following adjustment for age, BMI, smoking, alcohol, refraction, CKD, IHD, CVA and diabetes mellitus (Model 2), and medications used at a frequency >5% within the cohort (ACE inhibitors, aspirin, beta blockers, calcium channel blockers, corticosteroids, diuretics, NSAIDs and statins [Model 3]) and fellow vessel (Model 4), hypertension status remained significantly associated with arteriolar vessel caliber (Table 3: *P* = 0.002; effect size = -2.16 μm; CI: -3.51, -0.81). In an unadjusted analysis, individuals with hypertension had significantly narrower CRVE (*P* = 0.04; effect size = -2.26 μm; CI: -4.44, -0.08) compared to those without hypertension, although this was no longer significant following adjustment for other covariates (Table 3).

**Table 3:** Difference in mean retinal vascular caliber and fractal dimension between those with and without hypertension and CKD before and after adjustment for confounders.

Dependent variable	Model 1		Model 2		Model 3		Model 4	
	**Hypertension Coefficient (95% CI)**	***P***	**Hypertension Coefficient (95% CI)**	***P***	**Hypertension Coefficient (95% CI)**	***P***	**Hypertension Coefficient (95% CI)**	***P***
CRAE	-3.62 (-5.11, -2.14)	**<0.001**	-3.12 (-4.68, -1.56)	**<0.001**	-3.06 (-4.78, -1.35)	**<0.001**	-2.16 (-3.51, -0.81)	**0.002**
CRVE	-2.26 (-4.44, -0.08)	**0.04**	-1.54 (-3.82, 0.73)	0.18	-2.13 (-4.63, 0.37)	0.094	0.63 (-1.34, 2.61)	0.53
Fractal Dimension	-0.014 (-0.021, -0.007)	**<0.001**	-0.006 (-0.013, 0.002)	0.14	-0.006 (-0.014, 0.003)	0.17	NA	
	**CKD Coefficient (95% CI)**	***P***	**CKD Coefficient (95% CI)**	***P***	**CKD Coefficient (95% CI)**	***P***	**CKD Coefficient (95% CI)**	***P***
CRAE	-0.87 (-2.41, 0.67)	0.26	0.81 (-0.74, 2.37)	0.31	0.72 (-0.86, 2.30)	0.37	-0.12 (-1.37, 1.12)	0.85
CRVE	-0.27 (-2.52, 1.98)	0.81	2.07 (-0.20, 4.34)	0.07	1.98 (-0.32, 4.28)	0.09	1.33 (-0.47, 3.14)	0.15
Fractal Dimension	-0.005 (-0.013, 0.002)	0.16	0.005 (-0.002, 0.013)	0.19	0.005 (-0.003, 0.012)	0.23	NA	

Model 1- Unadjusted; model 2: adjusted for age, BMI, smoking status, alcohol, refraction, diabetes mellitus, chronic kidney disease or hypertension, ischemic heart disease, and cerebrovascular accident; model 3: adjusted for model 2 covariates and medications used at a frequency >5% within the cohort (angiotensin converting enzyme inhibitors, aspirin, beta blockers, calcium channel blockers, corticosteroids, diuretics, nonsteroidal anti-inflammatory drugs and statins).; model 4: adjusted for model 3 covariates and fellow vessel caliber. CRAE: central retinal arteriolar equivalent; CRVE: central retinal venular equivalent; 95% CI: 95% confidence intervals. NA: not applicable.

The mean D_*F*_ of participants with hypertension (1.413 +/- 0.062) was significantly sparser compared to those without hypertension (1.427 +/- 0.062) in an unadjusted analyses (-0.014: CI = -0.021, -0.007; *P* <0.001) although the significance of this association was not maintained after adjustment for potential confounders (Table 3).

### Chronic Kidney Disease

In both unadjusted and adjusted analyses, no significant differences were found between CRAE or CRVE by CKD status (Table 3). The mean D_*F*_ in participants with CKD was 1.417 +/- 0.061 and 1.423 +/- 0.063 in participants without CKD. No significant difference in mean D_*F*_ was detected between those with and without CKD in both adjusted and unadjusted analyses (Table 3).

## Discussion

In this study, we have demonstrated a significant narrowing of the retinal arterioles in association with hypertension (*P* = 0.002; effect size = -2.16 μm; CI: -3.51, -0.81) in white Irish nuns after adjustment for potential confounding variables. Retinal arteriolar narrowing is an established feature associated with hypertension, in which rising blood pressure triggers an auto-regulatory response resulting in increased arteriolar tone, narrowing of the pre-capillary arterioles and an increase in peripheral vascular resistance. The rise in peripheral vascular resistance can contribute to further elevation of blood pressure, and a detrimental process ensues [54].

This ‘*remodelling*’ of the retinal microvasculature has been highlighted in various population based studies and our study supports a recent meta-analysis [28], where narrower arterioles at baseline were associated with a 1.12 mmHg increase in SBP over 5 years. Ding and colleagues suggested that visualization of retinal vessels could quantify systemic microvascular dysfunction and provided strong evidence that these vascular changes actually preceded the development of hypertension [28]. These findings suggest the need for improved prevention and treatment strategies for hypertension targeted to optimize microvasculature structure, function, and tissue perfusion, with the aid of appropriate therapeutics combining vasodilatory, anti-oxidative, and anti-inflammatory properties [55].

Our study failed to detect any association between retinal venular caliber and hypertension, in contrast to a recent meta-analysis which reported venular widening is associated with hypertension [28]. Notably, other reports have shown retinal venular widening to be related to systemic inflammation, measures of atherosclerosis, and metabolic abnormalities [56]. Thus, findings related to the retinal venules are interesting as wider retinal venular caliber has not been considered to be a sign of hypertensive retinopathy. Further investigations of these relationships are therefore warranted in well-defined prospective cohorts. We also failed to detect any association between fractal dimension and hypertension. Other studies have reported a significant reduction in retinal vascular fractal dimension in association with hypertension [57, 58] suggesting a reduced or suboptimal retinal microvascular architecture as a result of an impaired and less efficient blood transportation system [51].

Given that the retinal vasculature is readily accessible and suitable for direct, non-invasive and repeated measurement, detection of subtle early microvascular changes prior to clinically significant events is possible, although it is unclear whether subsequent renal dysfunction is associated with abnormal retinal microcirculation in patients. Recent improvements in both digital retinal photography and imaging technology have enabled better characterization of retinal parameters. Animal studies have shown that microvascular injury contributes to the development and progression of CKD and conversely, reduced kidney function (decreased glomerular filtration rate) can lead to end-organ microvascular damage [59].

In our analysis, we failed to detect any association between CKD and retinal vascular parameters. The results published from other studies investigating the association between retinal vessel caliber and renal function have proved inconclusive, and findings relating to the global geometrical retinal vessel measurements are sparse in white cohorts. To the best of our knowledge there is only one previously published cross-sectional [39] and case-control [45] investigation on retinal fractal analysis and CKD, with no prospective data available. Retinal vascular caliber changes were not associated with renal functional decline in the Cardiovascular Health Study [36] or the Beaver Dam Eye Study [44], while in the Atherosclerosis Risk in Communities Study, both retinal arteriolar and venular narrowing were reported to be associated with a 6-year change in serum creatinine [35]. More recently, others have also reported narrowing of the retinal arterioles in association with CKD, independent of diabetes and hypertension, in an Asian population [37]. Sng and colleagues showed that a suboptimal layout of the retinal microvasculature was associated with CKD in a cross-sectional study and deviations from an optimal fractal dimension were related to certain disease processes [45], although we failed to replicate their finding.

Despite these inconsistencies, this relationship is of interest, as the retinal and renal microvasculature circulatory systems share comparable anatomical and physiological properties and common systemic microvascular processes may underlie the development of microvascular damage in both the eye and the kidney [33–34]. A better understanding of the underlying pathogenesis and risk predictors associated with CKD is essential for further development of improved preventative strategies.

The strengths of this study include the relatively large sample size, a high proportion of gradable digital retinal images, and masked evaluation of retinal vascular parameters by a trained grader using a semi-automated computer based technique [49]. We adhered to a standardized collection of data on potential confounders including anthropometric factors in a well-characterized cohort, and the relative uniformity of the nun’s backgrounds and lifestyles minimized further potential confounding, providing an opportunity for a more detailed examination of lifestyle and environmental factors that may contribute to the etiology of CKD, hypertension and evaluation of the retinal microvascular architecture. We have also used detailed participant prescription medication use to minimize potential confounding. Many previous studies have failed to adjust for the potential confounding effects of prescribed medication, particularly those with vasodilatory effects. The Cardiovascular Health Study did control for ACE Inhibitor use only [36] and a small case control study controlled for antihypertensive medication such as renin-angiotensin system blockers and calcium channel blockers [43]. Our study has sought to address the effects of such medication that may modify the retinal microvascular parameters measured. Furthermore, SIVA-FA is less likely to be adversely affected by artefacts, particularly those originating from media opacities, compared to the IRIS-Fractal software. This may arise as a consequence of the different vessel detection algorithms used in both software packages as IRIS-Fractal’s vessel line tracing algorithm also considers vessel caliber while SIVA-FA uses a skeletonized vessel tracing approach independent of vessel width [50]. Additionally, our study sample of white Irish nuns with minimal smoking (4% ever smoked), alcohol consumption (8% any alcohol), diabetes (3%) and heart disease (10%) had fewer of the potential confounding variables present in other studies, highlighting this novel population as a unique group to study in terms of a ‘healthy model of aging’.

Limitations of our study include its cross-sectional design, which did not let us determine whether retinal vascular changes observed, precede or occur as a consequence of hypertension. However, it could be hypothesized that changes in vessel caliber precede the development of hypertension and are further compounded by its presence [40]. Furthermore, certain data which may affect retinal vessel caliber including intraocular pressure [49] were unavailable. The glomerular filtration rate (GFR) is the best measure of kidney function in health and disease, although we did not examine the GFR directly, but estimated it from serum creatinine levels. In addition, artefacts generated from choroidal vessels, peripapillary atrophy, pigmentary abnormalities and reflection from the nerve fiber layer during fractal analysis required manual intervention which could potentially introduce some systematic bias. We also used a single blood pressure measurement with self-report to identify participants classified as hypertensive. It is recognized that single clinical measurements may correlate poorly with blood pressure captured in other settings, prompting recommendations for 24-hour ambulatory monitoring to provide a more accurate diagnosis of hypertension and reducing the problem of “white-coat” hypertension [60]. While convent or religious orders may not truly reflect the general population, they nevertheless represent an excellent opportunity to study a well-characterized model of ‘healthy aging’.

In summary our cross-sectional study of aged white Irish Nuns has shown that retinal arteriolar narrowing is significantly associated with hypertension but that no significant association between retinal vascular parameters and CKD was determined. These findings may provide support for earlier and tailored prevention and treatment options in hypertension that ultimately optimize the structure, function and viability of the microvasculature, providing additional clinical utility for the treatment of microvascular disease.
